# Bamboo Stems (*Phyllostachys nigra* variety henosis) Containing Polyphenol Mixtures Activate Nrf2 and Attenuate Phenylhydrazine-Induced Oxidative Stress and Liver Injury

**DOI:** 10.3390/nu11010114

**Published:** 2019-01-08

**Authors:** Ji Hye Yang, Moon-Hee Choi, Chang-Su Na, Sam Seok Cho, Jae Hoon Kim, Sae Kwang Ku, Il Je Cho, Hyun-Jae Shin, Sung Hwan Ki

**Affiliations:** 1 Laboratory of Toxicology, College of Pharmacy, Chosun University, Gwangju 61452, Korea; uranus2k@nate.com (J.H.Y.); samseok7@gmail.com (S.S.C.); 93_kjh@naver.com (J.H.K.); 2 College of Korean Medicine, Dongshin University, Naju, Jeollanam-do 58245, Korea; nakugi@hanmail.net; 3 Department of Biochemical and Polymer Engineering, Chosun University, Gwangju 61452, Korea; aamoony1222@naver.com; 4 RC-HCLD, College of Korean Medicine, Daegu Haany University, Gyeongsan, Gyeongsangbuk-do 38610, Korea; gucci200@hanmail.net (S.K.K.); skek023@dhu.ac.kr (I.J.C.)

**Keywords:** bamboo stems, PN3, Nrf2, iron overload, liver injury

## Abstract

This study was designed to investigate the hepatoprotective effect of bamboo stems using in vitro and in vivo experimental liver damage models. Ethyl acetate fraction of 80% ethanol extract of *Phyllostachys nigra* stem (PN3) containing polyphenols had a higher *NQO1-ARE* reporter gene activity as monitored by the activity of the NF-E2-related factor (Nrf2) antioxidant pathway in cells in comparison to extracts from other species and under other conditions. The Nrf2 was translocated from the cytosol to the nucleus in response to PN3, followed by induction of the Nrf2 target gene expression, including *HO-1*, *GCL*, and *NQO-1* in HepG2 cells. Phosphorylation of Nrf2 in HepG2 cells was enhanced in PN3, which was mediated by PKCδ, ERK, and p38 MAPK. Consequently, PN3 inhibited arachidonic acid (AA) + iron-induced reactive oxygen species generation and glutathione depletion, and, thus, highlighted their role in cytotoxicity. Treatment with major polyphenols of PN3, including catechin, chlorogenic acid, caffeic acid, and *p*-coumaric acid, also improved AA + iron-mediated oxidative stress and, thus, improved cell viability. Treatment with phenylhydrazine in mice, i.e., the iron overload liver injury model, increased plasma alanine aminotransferase and aspartate aminotransferase levels and changed histological features in mice—a response that was almost completely blocked by PN3 administration. Moreover, PN3 extract mitigated phenylhydrazine-induced oxidative stress and inflammatory responses. Conclusively, PN3 can exert a hepatoprotective effect against iron overload-induced acute liver damage due to its antioxidant properties.

## 1. Introduction

Liver diseases make a large contribution to the global burden of morbidity and mortality. It is well-established that patients with chronic liver diseases are much more likely to accumulate a large amount of iron in the hepatic parenchyma and in the reticuloendothelial cells [1]. Liver is the main organ responsible for iron homeostasis and, thus, several liver diseases are markedly influenced by iron overloads in the liver [2]. Higher iron levels are present not only in patients with hereditary hemochromatosis [3], but also in those with alcoholic liver disease [4], nonalcoholic fatty liver disease [5], and chronic hepatitis C viral infection [6].

Several studies indicate that oxidative stress, caused by elevation of reactive oxygen species (ROS), might play a role in the pathogenesis of iron-induced liver injury. Through Haber-Weiss and Fenton reactions, iron produces ROS, including hydroxyl radicals, and consequently, leads to oxidative damage of intracellular macromolecules; this eventually results in lipid peroxidation of the cell membrane [7]. Several reports show that iron-induced oxidative damage increases lysosomal membrane fragility [8], causes a decrease in cytochrome c oxidase activities [9], and damages the mitochondria [10]. Previously, we and other research groups, have reported that arachidonic acid (AA) synergizes the efficacy of iron to increase oxidative stress and mitochondrial dysfunction, thereby eliciting cellular stress and cytotoxicity in hepatocytes [11,12]. Hence, an in vitro experimental co-treatment model of AA and iron was employed to explore the potential of candidate drugs and mechanisms of action for the therapy of oxidative stress-mediated liver injury [12,13].

The NF-E2-related factor (Nrf2), a master transcription factor in the antioxidant defense system, upregulates a variety of downstream target genes responsible for cytoprotection [14]. Activation of Nrf2 has been implicated as a potential therapeutic target for the prevention and treatment of liver disease [15]; Nrf2 is known to be activated in response to oxidative stress, whereby it upregulates its target genes by binding to the antioxidant response element (ARE), which is a *cis*-acting enhancer sequence in their promoters. Its activity is mainly regulated through its interaction with its cytoplasmic inhibitor, Keap1 (Kelch-like ECH-associated protein 1), which directs ubiquitin proteasome-mediated degradation [16]. There is an increasing amount of evidence regarding other mechanisms of Nrf2 regulation, including direct phosphorylation of Nrf2 by several protein kinases [17].

Polyphenols are abundant micronutrients in our diet, and the evidence of their health effect is emerging [18]. These compounds may be classified into four different groups as a function of the number of phenol rings that they contain and of the structural elements that bind these rings to one another; phenolic acids, flavonoids, stilbenes, and lignans [18]. Polyphenols, a class of plant and fungus secondary metabolites, are ubiquitously distributed in relatively high concentrations in all foods and plants [19]. Polyphenols have been shown to have a wide range of biological and pharmacological activities in in vitro studies, for example, positive effects against oxidative stress, inflammatory responses, insulin resistance, apoptosis, cellular proliferation, and cancer, thereby showing its potential for hepatoprotective effects [20,21]. Although there is ongoing research into the potential health benefits of individual polyphenols, neither the Food and Drug Administration nor the European Food Safety Authority has approved any health claim for polyphenols. Thus, in vitro and in vivo studies are still required to support the claims for the safety and efficacy of flavonoids for nutraceutical and functional food applications.

Bamboo is a member of the Poaceae plant family and is used as a traditional medicine for the treatment of diverse symptoms, including cough, fever, and leprosy for over 1000 years. Although, it has been well-studied that bamboo leaves and shoot oil have anti-oxidant, anti-inflammatory, anti-microbial, anti-ulcer, anti-diabetic, anti-fertility, and anti-hypertensive effects, the exact pharmacological properties of bamboo stems remain elusive [22]. We previously reported that ethyl acetate fraction of 80% ethanol (EtOH) extract from domestic bamboo stems of *Phyllostachys nigra* var. henosis (PN3) contain the highest known content of polyphenol compounds (catechin, chlorogenic acid, caffeic acid, and *p*-coumaric acid); this extract also has an inhibitory effect on melanogenesis, as well as an antioxidant capacity [23]. However, the potential of bamboo stem extract for other pharmacological actions, including hepatoprotective effect, associated with its anti-oxidative efficacy has not been described yet.

In this study, we investigated the hepatoprotective effect of bamboo stems containing polyphenol mixtures that are beneficial to health. To evaluate the cytoprotective effect of bamboo stems extract and its major polyphenols, we used AA + iron-induced oxidative injury in vitro model. Moreover, mice were treated with phenylhydrazine (PHZ) to induce acute liver injury associated with iron overload caused by hemolysis and administered with bamboo stems to investigate its hepatoprotective effects in vivo.

## 2. Materials and Methods

### 2.1. Materials

Arachidonic acid (AA), PD98059, SB203580, and SP600125, were obtained from Calbiochem (Billerica, MA, USA), while NAD(P)H quinone oxidoreductase-1 (NQO-1), lamin A/C, and caspase-3 antibodies were provided by Cell Signaling (Danvers, MA, USA). Bad antibody was obtained from BD Bioscience (Becton Dickinson Biosciences, Mountain View, CA, USA). Antibodies against Nrf2, phospho-Nrf2, Bax, Bcl-xL, and Poly (ADP-ribose) polymerase (PARP) were obtained from Santa Cruz Biotechnology (Santa Cruz, CA, USA). Hemoxygenase (HO)-1 antibody was purchased from Enzo Life Sciences (Plymouth Meeting, PA, USA) and glutamate cysteine ligase (GCL) antibody from Abcam (Cambridge, MA, USA). Rottlerin, compound C, phenylhydrazine (PHZ), 3-(4,5-Dimethylthiazol-2-yl)-2,5-diphenyltetrazolium bromide (MTT), 2′,7′-dichlorodihydrofluorescein diacetate (DCFH-DA), dimethyl sulfoxide, and β-actin antibody were provided by Sigma (St. Louis, MO, USA). The iron-NTA complex (1:3 Fe/NTA) was prepared as described previously [24].

### 2.2. Preparation of Bamboo Stem Extracts

Previously, we succeeded in extracting domestic bamboo stems using water and EtOH; the extracts were then separated into hydrophilic and hydrophobic fractions with methanol (MeOH), *n*-hexane, and ethyl acetate [23]. Stems from two species of bamboo, *P. nigra* var. henonis (Mitford) Rendle (PN) and *P. bambusoides* Siebold and Zucc (PB), were used in this study. The bamboo stems were purchased from a local supplier in Damyang City (35°20′44.6″N 126°59′55.5″E), Korea, during the summer season of 2017. Powdered bamboo stems were extracted with water at 1.5 atm and 121 °C for 90 min (autoclaving). Various concentration of EtOH (50%, 80%, and 100%) were used to extract bamboo stems for 3–4 days at room temperature (25 ± 2 °C). Boiled water was also used to prepare the extract at 100 ± 1 °C for 24 h (water heating). The ten extracts mentioned above (five from PN and five from PB) were vacuum-concentrated, re-suspended with 50% MeOH, and partitioned with *n*-hexane and EtOAc. The final EtOAc layers from each solvent and method were named as autoclaving (PB1 and PN1), 50% EtOH (PB2 and PN2), 80% EtOH (PB3 and PN3), 100% EtOH (PB4 and PN4), and boiling water (PB5 and PN5). The names of the five extracts are the same as those used in the previous report [23].

### 2.3. HPLC Analysis of Bamboo Stem Extracts

Among five extracts prepared as above, PN3 was selected and analyzed quantitatively by high-performance liquid chromatography (HPLC). The Shimadzu HPLC system (Japan) consisted of a LC-20AD pump, a diode array detector (SPD-20A), and a Shim-Pack GIS-ODS C_18_ column (4.6 × 150 mm, 5 µm). The mobile phases consisted of solvent (A), 0.3% (*v*/*v*) acetic acid in water; and solvent (B), 0.3% (*v*/*v*) acetic acid in MeOH. The gradient elution program was as follows: 0–80 min, 10–60% B. The flowrate was 0.7 mL/min. The column temperature was 40 °C, and the absorption was measured at 254 nm. Samples consisting of 30 µL were injected sequentially. The compounds were identified by comparing the retention times of standard materials.

### 2.4. Animals

The protocols for animal studies were approved by the Animal Care and Use Committee of Chosun University. Male ICR mice (six weeks old) were provided by Oriental Bio (Sung-nam, Korea) and acclimatized for one week. Mice (*N* = 10/group) were housed at 20 ± 2 °C with 12 h light/dark cycles and a relative humidity of 50 ± 5% under filtered, pathogen-free air, with food (Purina, Korea) and water available *ad libitum*. Ethyl acetate fraction of 80% EtOH extract of *P. nigra* stem (PN3) (250–500 mg/kg) was orally administered to the mice for four days. Before the mice were sacrificed, to induce iron accumulation and liver injury, PHZ (60 mg/kg/day, i.p.) was injected into the mice for the last two days before sacrifice (1 h after PN3 treatment). Mice were sacrificed 24 h after the second PHZ injection.

### 2.5. Blood Chemistry

Method of analyzing blood chemistry was as described previously [25]. Plasma alanine aminotransferase (ALT) and aspartate aminotransferase (AST) in mice serum were measured with commercial kits (Asan Pharmaceutical, Seoul, Korea).

### 2.6. Histopathology

Samples from the liver were separated and fixed in 10% neutral buffered formalin, followed by being embedded into paraffin, sectioned (3–4 µm), and stained with hematoxylin and eosin (H and E). Subsequently, the histopathological profiles of each sample were observed under light microscope (Carl Zeiss, Oberkochen, Germany). The changes in the histopathology of hepatic tissues were observed under general H and E staining in the present study. To observe more detailed changes, the percentage of degenerative regions in hepatic parenchyma (%/mm^2^) showing centrolobular necrosis, congestion, and inflammatory cells on hepatic lobules, the number of hepatocytes showing degenerative changes (necrosis, acute cellular swelling, and severe fatty changes) per 1000 hepatocytes, and the number of inflammatory cells in hepatic parenchyma (%/mm^2^) were recorded. In addition, the extents of 4-hydroxynonenal (4-HNE, Abcam, Cambridge, UK), cyclooxygenase (COX)-2 (Cayman Chemical., Ann Arbor, MI, USA), and ferritin (Abcam, Cambridge, UK) immunoreactivities were also observed by Avidin-biotin-peroxidase complex (Vector Lab., Burlingame, CA, USA) using immunehistochemical methods in the restricted-view fields of hepatic parenchyma with positive cells/1000 hepatocytes, respectively.

### 2.7. Assay of ROS Generation

Ten milligrams of mice liver tissue was homogenized in 1 mL of PBS and DCFH levels were determined as an index of the peroxide production as previously reported [26]. Briefly, 50 µL of freshly prepared liver homogenate was mixed with 4.85 mL of 100 mM potassium phosphate buffer (pH 7.4) and incubated with DCFH-DA at a final concentration of 5 μM for 15 min at 37 °C. After centrifuging at 10,000× *g* for 10 min at 4 °C, the pellet was suspended in 5 mL of potassium phosphate buffer (pH 7.4) on ice and incubated for 60 min at 37 °C as described previously [26]. In order to investigate the effect of PN3 extract on AA + iron-induced ROS production, HepG2 cells were incubated with AA (15 h) + iron (1 h) and/or 50–100 µg/mL PN3. Cells were stained with 10 µM DCFH-DA for 30 min at 37 °C. The collected cells were then washed twice with a wash buffer, and H_2_O_2_ generation was determined using a fluorescence-detecting microplate reader (Jemini, Molecular Device, Sunnyvale, CA, USA) at excitation/emission wavelengths of 485/530 nm. The production of ROS was normalized as per the protein concentration of each treated sample and was defined relative to the vehicle-treated control.

### 2.8. Determination of GSH Content

Method of the determination of GSH content in mice liver homogenates or HepG2 cells has been described previously [26]. Briefly, the samples were lysed in a buffer containing 5% metaphosphoric acid to precipitate proteins. The cell lysates or homogenates were centrifuged at 10,000× *g* for 10 min, and the supernatants were used to measure GSH concentrations according to the manufacturer’s manual. Absorbance was measured using a microplate reader (410 nm).

### 2.9. Primary Hepatocyte Isolation

The ICR mice were anesthetized, and the portal vein was cannulated under aseptic conditions. The liver was perfused in situ with Ca^2+^-free Hank’s balanced saline solution (HBSS) at 37 °C for 5 min as previously described [26]. Livers were then perfused for 20 min with HBSS containing 0.05% collagenase and Ca^2+^ at a perfusion flow rate of 10 mL/min. After perfusion, the livers were minced gently with scissors and suspended in sterilized PBS. The cell suspension was then filtered through a cell strainer and centrifuged at 50× *g* for 5 min to separate parenchymal and nonparenchymal cells. Isolated hepatocytes were seeded into collagen-coated plates and cultured in Dulbecco’s Modified Eagle Medium (DMEM) containing 50 units/mL penicillin/streptomycin, along with 10% Fetal Bovine Serum (FBS), as described previously [27].

### 2.10. Cell Culture

HepG2 cells obtained from ATCC (American Type Culture Collection, Manassas, VA, USA) were maintained in DMEM containing 50 units/mL penicillin/streptomycin with 10% FBS at 37 °C in a humidified 5% CO_2_ atmosphere.

### 2.11. Immunoblot Analysis

Protein extraction, subcellular fractionation, SDS-polyacrylamide gel electrophoresis, and immunoblot analyses were performed according to previously published procedures [28]. Briefly, the samples were separated using 7.5% and 12% gel electrophoresis; they were then electrophoretically transferred to nitrocellulose paper. The nitrocellulose paper was first incubated with the indicated primary antibodies, followed by incubation with horseradish peroxidase-conjugated secondary antibody. Immunoreactive proteins were visualized by ECL chemiluminescence detection (Amersham Biosciences, Buckinghamshire, UK). The equivalency of protein loading and the integrity of nuclear fractionation were verified by β-actin or lamin A/C immunoblotting, respectively.

### 2.12. Cytotoxicity Assay

To measure cell viability, cells were plated in 48-well plates and treated with chemicals for 24 h; the viable cells were then stained with MTT (0.2 mg/mL, 4 h) as previously reported [25]. The media were then removed, and formazan crystals produced in the wells were dissolved with the addition of 200 µL of dimethyl sulfoxide. Absorbance at 540 nm was measured using a microplate reader (Spectramax, Molecular Device, Sunnyvale, CA, USA). Cell viability was defined relative to untreated control (i.e., viability (% control) = 100 × (absorbance of treated sample)/(absorbance of control)).

### 2.13. Luciferase Assay

To provide rapid and reproducible results for the Nrf2 activity, we established a HepG2 cell line that had been stably transfected with an *NQO1-*ARE firefly luciferase construct; this construct contained 3 tandem repeats of the ARE in the 5′-upstream region of *NQO1*. The activity of firefly luciferase was examined by luciferase assay reagent II (Promega, Madison, WI, USA) according to the manufacturer’s instructions.

### 2.14. Statistical Analysis

One-way analysis of variance (ANOVA) was used to assess the statistical significance of differences among the treatment groups. The Newman–Keuls test was used to determine the significance of differences between the means of multiple groups. Results are expressed as mean standard deviations (S.D.).

## 3. Results

### 3.1. Nrf2 Activation by PN3

**Figure 1:**
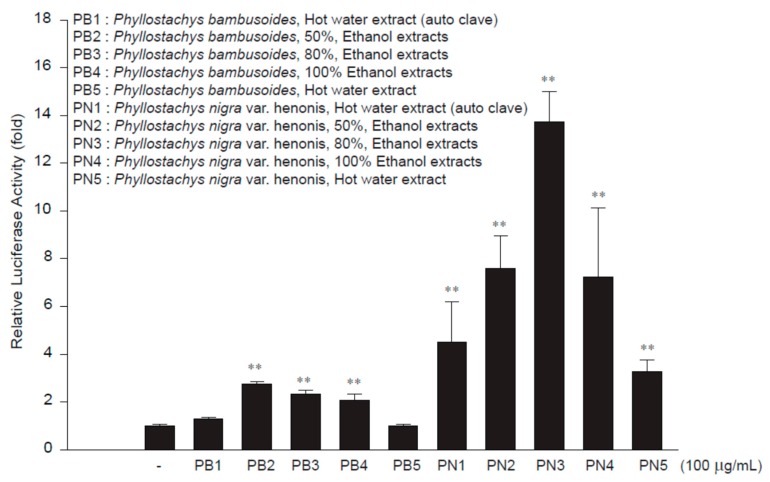
NAD(P)H quinone oxidoreductase-1 (NQO1)-antioxidant response element (ARE) luciferase activity of bamboo stem extract in HepG2 cells. *NQO1-ARE* luciferase activity was measured on the lysates of HepG2 cells that were stably transfected with the *NQO1-ARE* luciferase construct exposed to extract from *P. bambusoides* (PB) and *P. nigra* var. henonis (PN) for 12 h. Data represent the mean ± S.D. of three replicates; ** *p* < 0.01, significant versus vehicle-treated control.

First, we performed *NQO-1/*ARE luciferase assay to compare the antioxidant effects of the two domestic bamboo species, *P. bambusoides* (PB) and *P. nigra* var. heronis (PN), on Nrf2 activation. Extracts had no cytotoxic effect in HepG2 cells at concentrations of up to 100 μg/mL (Supplementary Figure S1). However, PN increased luciferase activity as compared to PB; also, the ethyl acetate fraction of 80% EtOH extract from the stems of *P. nigra* var. henosis (PN3) had the highest ARE luciferase activity among all fractions (Figure 1). Thus, we examined whether PN3 can exert anti-oxidative and cytoprotective effects in HepG2 cells, human hepatocyte-derived hepatocellular carcinoma cell line.

**Figure 2:**
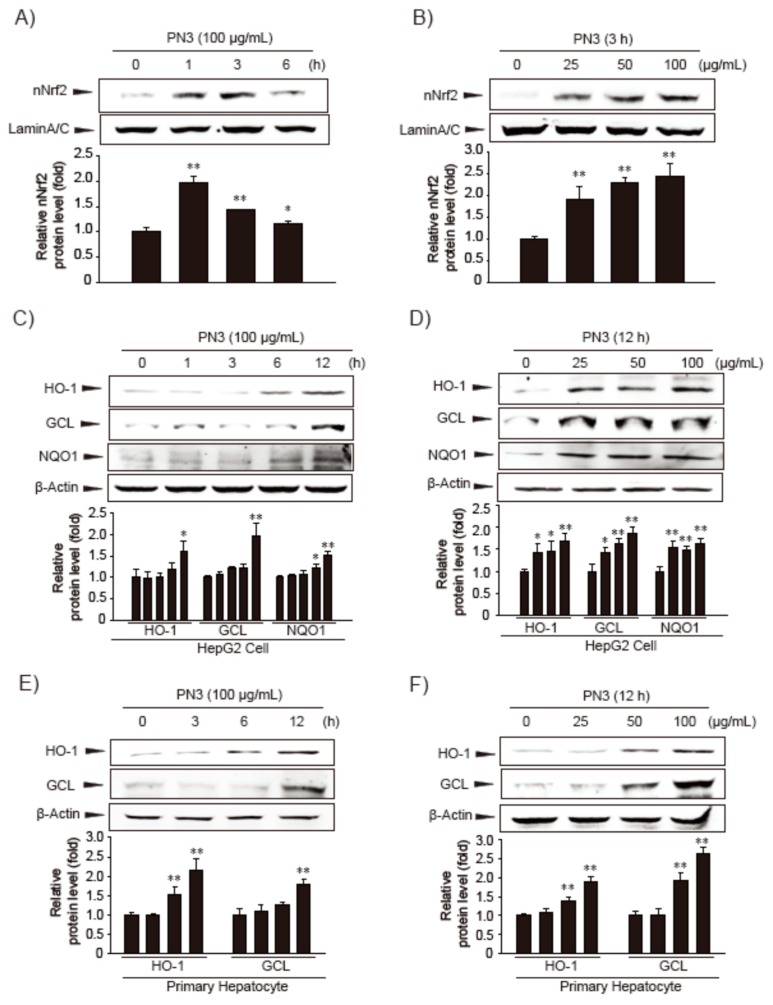
The effect of the ethyl acetate fraction of 80% EtOH extract from the stems of *P. nigra* var. henosis (PN3) on NF-E2-related factor (Nrf2) activation and Nrf2 target gene expression. (**A**) Time courses of nuclear Nrf2 (nNrf2) translocation in HepG2 cells treated with 100 µg/mL PN3. The Nrf2 protein was immunoblotted from the nuclear fractions of cells that had been incubated with PN3 for 1 to 6 h. (**B**) Varying concentrations of PN3 for 3 h on nuclear Nrf2 translocation in HepG2 cells. Nuclear Nrf2 protein was immunoblotted from cells treated with PN3 (25–100 µg/mL). (**C**) HepG2 cells were treated with 100 µg/mL PN3 for 1 to 12 h. The proteins, HO-1, GCL, and NQO1, were immunoblotted from the lysates of cells. (**D**) The expression of Nrf2 target genes in HepG2 cells treated with indicated concentrations of PN3 for 12 h. (**E**,**F**) The effect of PN3 on the expression of Nrf2 target genes in mouse primary hepatocytes. Primary hepatocytes were incubated for varying times (3–12 h) and at different concentrations (25–100 µg/mL) of PN3. Data represent the mean ± S.D. of 3 replicates; * *p* < 0.05, ** *p* < 0.01, significant versus vehicle-treated control.

Next, we treated HepG2 cells with 100 µg/mL PN3 extract for 0–6 h and then investigated the effect of PN3 on the nuclear translocation of Nrf2 (Figure 2A). We found that nuclear Nrf2 levels increased and peaked after 1–3 h of PN3 treatment. Treatment with PN3 substantially increased the nuclear Nrf2 levels in a dose-dependent manner (Figure 2B). Subsequently, we analyzed the expression of Nrf2 target genes, such as heme oxygenase 1 (HO-1), glutamate cysteine ligase (GCL), and NAD(P)H quinone oxidoreductase (NQO)-1. Expression of the Nrf2 target genes was indeed induced by PN3 treatment in HepG2 cells (Figure 2C,D) and isolated primary hepatocytes from mice (Figure 2E,F).

### 3.2. Nrf2 Phosphorylation by ERK, P38 MAPKs, and PKCδ in PN3

**Figure 3:**
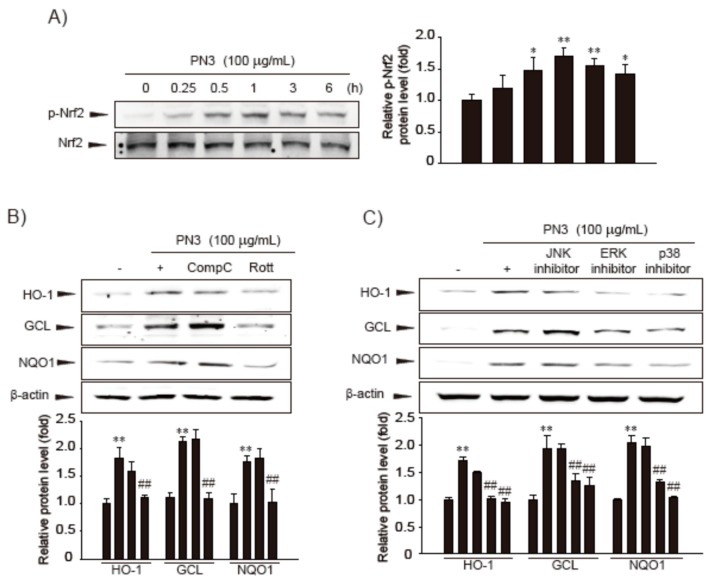
The effect of PN3 on Nrf2 activation. (**A**) Immunoblot analysis for phosphorylation of Nrf2 in HepG2 cells treated with PN3. Levels of Nrf2 phosphorylation were determined in the cell lysates that had been incubated with 100 μg/mL PN3 for 15 min to 6 h. Results were confirmed by repeated experiments. (**B**) HO-1, GCL, and NQO1 proteins were immunoblotted from cells treated with PN3 and/or AMPK or PKCδ inhibitor to analyze the effects of PN3. Cells were treated with 100 μg/mL PN3 in a concomitant treatment with 5 µM compound C (AMPK inhibitor, CompC) or 2.5 µM rottlerin (PKCδ inhibitor, Rott) for 12 h. (**C**) The effects of PN3 via the activations of MAPK on Nrf2 target gene expression. The HO-1, GCL, and NQO1 proteins were immunoblotted from cells treated with PN3 and/or MAPK inhibitors. Cells were treated with 100 µg/mL PN3 and/or MAPK inhibitors (each at a concentration of 10 μM) for 12 h. Data represent the mean ± S.D. of three replicates; * *p* < 0.05, ** *p* < 0.01, significant versus vehicle-treated control; ^##^ *p* < 0.01, significant versus PN3 alone.

Phosphorylation of Nrf2 by various protein kinases leads to its release from Keap1, which allows it to translocate into the nucleus, where it exerts gene transcription [17]. Treatment with PN3 for 0.25–6 h led to an increase in the phosphorylation of Nrf2, and the phosphorylation levels peaked after 0.5–3 h of PN3 treatment (Figure 3A). To identify the molecular mechanism causing the phosphorylation of Nrf2 by PN3, we examined which upstream kinases regulate the PN3-mediated phosphorylation of Nrf2 using chemical inhibitors. Treatment with PD98059 (an ERK inhibitor), SB203580 (a p38 MAPK inhibitor), or rottlerin (a PKCδ inhibitor), but not compounds C (an AMPK inhibitor) and SP600125 (a JNK inhibitor), attenuated HO-1, GCL, and NQO-1 induction by PN3 (Figure 3B,C). These observations support that PN3 activates Nrf2 and the expression of its target genes by activating ERK, p38 MAPK, or PKCδ.

### 3.3. Cytoprotective Effect of PN3

**Figure 4:**
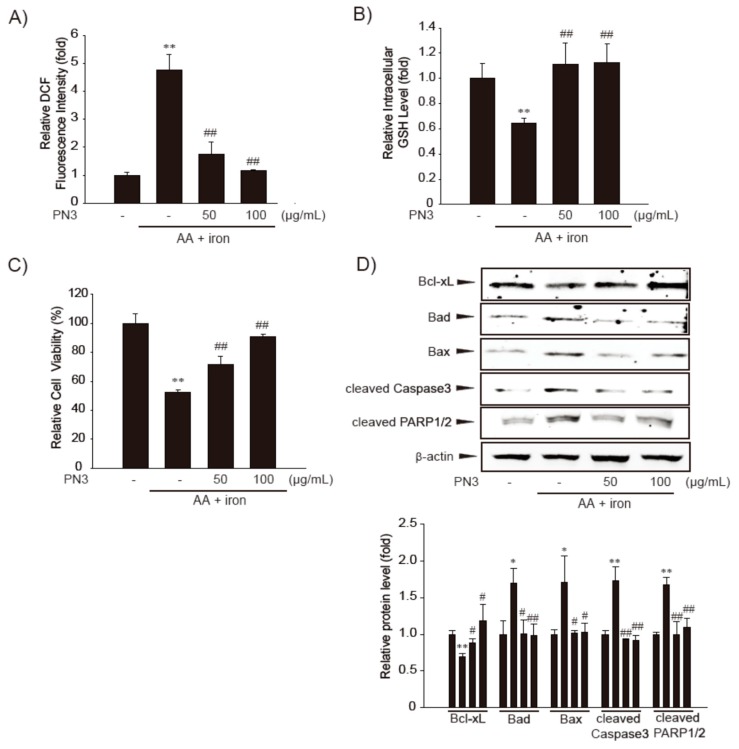
The cytoprotective effect of the PN3. (**A**) The effect of PN3 on AA + iron-induced ROS production. The HepG2 cells were pretreated with PN3 (50–100 µg/mL, for 1 h), and then exposed to AA (15 h) + iron (1 h). Cells were stained with 10 µM DCFH-DA for 30 min at 37 °C. Intracellular fluorescence intensities were measured using a fluorescence microplate reader. (**B**) The GSH concentrations were measured in the cell lysates that were treated with AA (15 h) + iron (2 h) and/or PN3 (50–100 µg/mL). (**C**) Cell viability. Cells were treated with AA (15 h) + iron (6 h) and/or 50–100 µg/mL PN3. The effect of PN3 on AA + iron-induced cell death was assessed using MTT assays. (**D**) Representative western blots exhibiting the levels of proteins involved in cell death in the presence or absence of AA (15 h) + iron (4 h) and/or 50–100 µg/mL PN3 (upper). The levels of Bcl-xL, Bad, Bax, cleaved caspase3, and cleaved PARP1/2 proteins in the cells as determined by scanning densitometry (lower). Data represent the mean ± S.D. of 3 replicates; * *p* < 0.05, ** *p* < 0.01, significant versus vehicle-treated control; ^#^ *p* < 0.05, ^##^ *p* < 0.01, significant versus AA + iron alone.

Next, we examined whether PN3 could block AA + iron-mediated oxidative stress and ameliorate cell damage in HepG2 cells. To evaluate the protective effects of PN3 against AA + iron-induced cell death, the cells were pretreated with PN3 for 1 h, followed by incubation with AA (10 µM) + iron (1 µM). Treatment with AA + iron increased ROS production, which was significantly reduced by PN3 pre-treatment (Figure 4A). In addition, AA + iron-induced depletion of intracellular glutathione levels was prevented by PN3 treatment (Figure 4B). Furthermore, cell viability was examined by colorimetric MTT assay. Pre-treatment with PN3 increased cell viability in AA + iron-treated cells in a dose-dependent manner (Figure 4C). To confirm the protective effect of PN3 on AA + iron-mediated apoptosis, we examined changes in the levels of apoptosis-related proteins. AA + iron decreased the level of Bcl-xL and increased the Bad and Bax levels, but these changes were prohibited by PN3 pretreatment (Figure 4D). Furthermore, PN3 was consistently observed to be capable of inhibiting increased cleavages of PARP and caspase-3 by AA + iron. These findings suggest that the inhibition of ROS production by PN3 can protect cells against oxidative stress-mediated cell death.

### 3.4. Effect of the Major Functional Constituents of PN3 in AA + Fe-Treated HepG2 Cells

**Figure 5:**
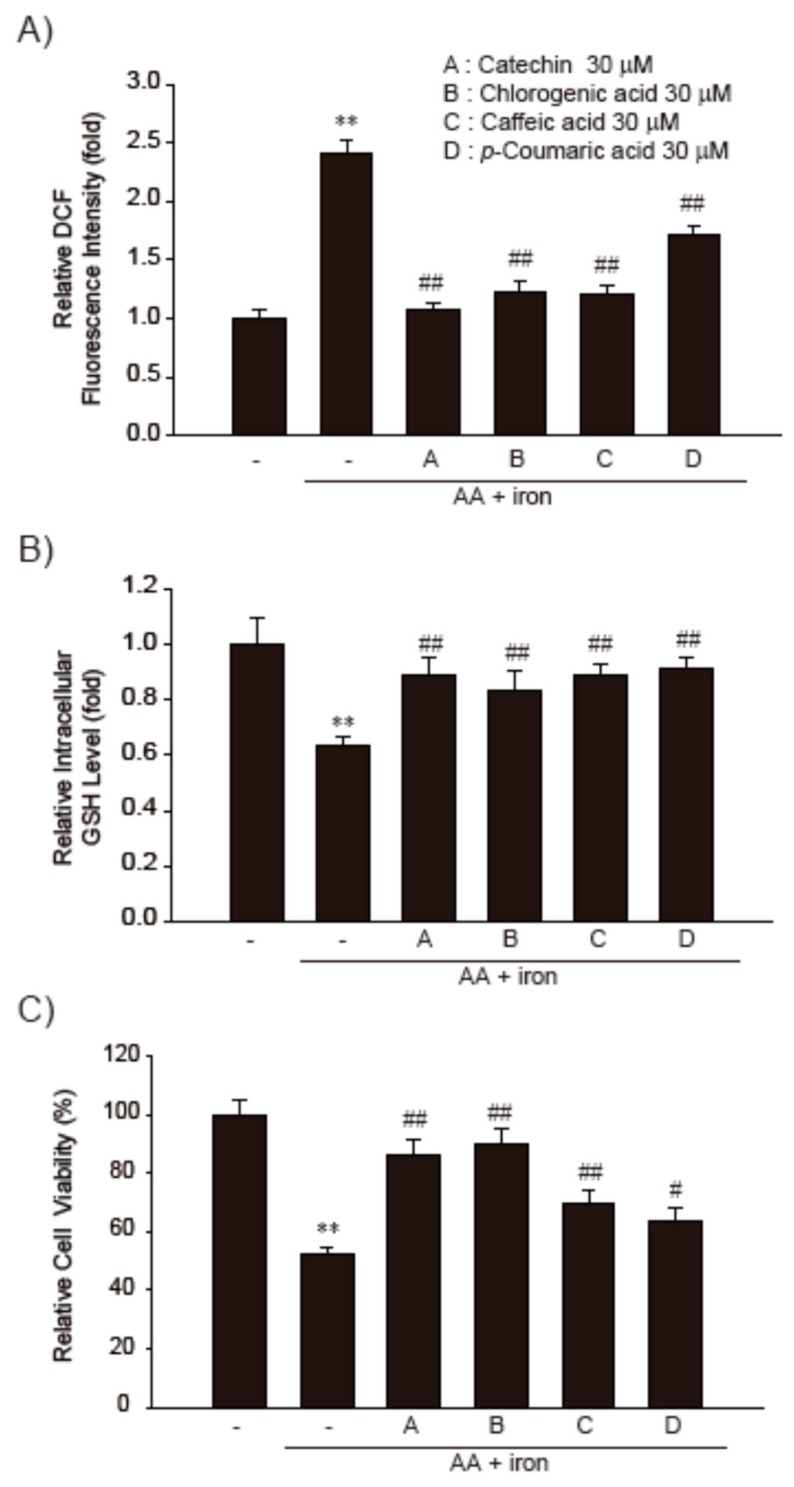
The cytoprotective effects of major polyphenols of the PN3. (**A**) HepG2 cells were incubated with AA (15 h) + iron (1 h) and/or 30 µM major polyphenols of PN3 (catechin, chlorogenic acid, caffeic acid, or *p*-coumaric acid). Cells were stained with 10 µM DCFH-DA for 30 min at 37 °C. Intracellular fluorescence intensities were measured using a fluorescence microplate reader. (**B**) The GSH concentrations were measured in the lysates of cells treated with AA (15 h) + iron (2 h) and/or 30 µM of major polyphenols of PN3. (**C**) Cell viability. Cells were treated with AA (15 h) + iron (6 h) and/or 30 µM major polyphenols of PN3 (catechin, chlorogenic acid, caffeic acid, or *p*-coumaric acid). Data represent the mean ± SD of three replicates; ** *p* < 0.01, significant versus vehicle-treated control; ^#^ *p* < 0.05, ^##^ *p* < 0.01, significant versus AA + iron alone.

We previously succeeded in identifying and characterizing the main functional components in PN3 extract. Like other bamboo extracts, PN3 extract contains many polyphenols, such as catechin (701.39 µg/g), chlorogenic acid (297.65 µg/g), caffeic acid (241.94 µg/g), and *p*-coumaric acid (193.11 µg/g) [23]. Therefore, we subsequently examined the major constituents of PN3 responsible for the inhibition of AA + iron-induced oxidative stress and cytotoxicity. These compounds showed no cytotoxicity on HepG2 cell lines even at their highest concentrations tested. We compared the relative prohibitory efficacies of the major polyphenols of PN3 on the AA + iron-induced cell death, ROS production, and intracellular GSH depletion in HepG2 cells. The production of ROS and GSH depletion by AA + iron were significantly inhibited by treatment with each of the polyphenols in PN3 extract (Figure 5A,B). Consistently, AA + iron-mediated cell viability reductions were markedly inhibited by treatment with these major polyphenols of PN3, although catechin and chlorogenic acid were observed to have a more potent cytoprotective effect than other compounds (Figure 5C). Our results indicate that the primary polyphenols of PN3 inhibit AA + iron-induced oxidative damage and cell death.

### 3.5. Effect of PN3 on PHZ-Induced Acute Liver Injury

**Figure 6:**
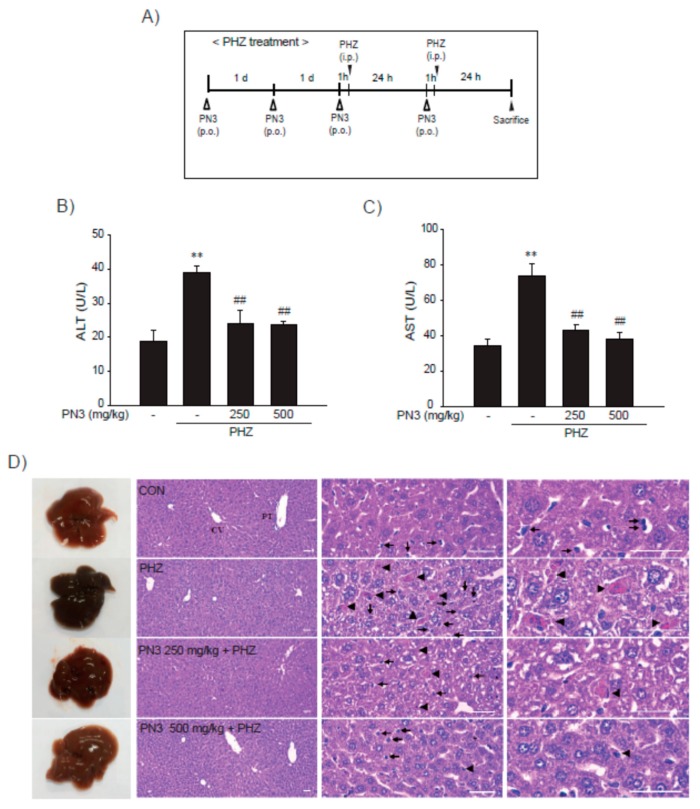
The effect of the ethyl acetate fraction of PN3 on phenylhydrazine (PHZ)-induced liver injury. (**A**) Study design. PN3 (250–500 mg/kg) were used to treat mice for 4 days via oral administration. To induce iron accumulation and liver injury, PHZ (60 mg/kg/day, i.p.) was injected into the mice for the last two days during PN3 administration before they were sacrificed. All mice were sacrificed 24 h after the second PHZ injection. (**B**,**C**) PN3 inhibited PHZ-induced liver injury. The levels of ALT (**B**) and AST (**C**) were determined using commercial kits. Data represent the mean ± S.D.; ** *p* < 0.01, significant versus vehicle-treated control; ^##^ *p* < 0.01, significant versus PHZ alone. (**D**) Representative histological sections of the liver. Samples were fixed in 10% neutral buffered formalin, embedded in paraffin, sectioned (3–4 µm), and then stained with H and E for general histological observations (scale bar = 120 µm). Arrow heads and arrows indicate representative degenerative hepatocytes and inflammatory cells, respectively. CV = central vein; PT = portal triad.

The PHZ induces acute liver injury by accumulation of iron in hepatic tissues [29,30,31]. To examine the hepatoprotective effect of PN3 in vivo, ICR mice were administered PN3 (250–500 mg/kg/day) for 4 days and were injected with PHZ (60 mg/kg/day) on days 3 and 4 (Figure 6A). Serum biochemistry results showed ALT and AST levels to have increased due to PHZ, indicating the presence of acute liver injury. On the other hand, oral administration of PN3 attenuated these increases (Figure 6B,C). Histopathological evaluations of liver tissues indicated that peripheral degenerative changes of hepatic lobules and inflammatory cell were increased due to PHZ. However, these PHZ-induced histological changes were markedly inhibited by PN3 (Figure 6D, Table 1).

**Figure 7:**
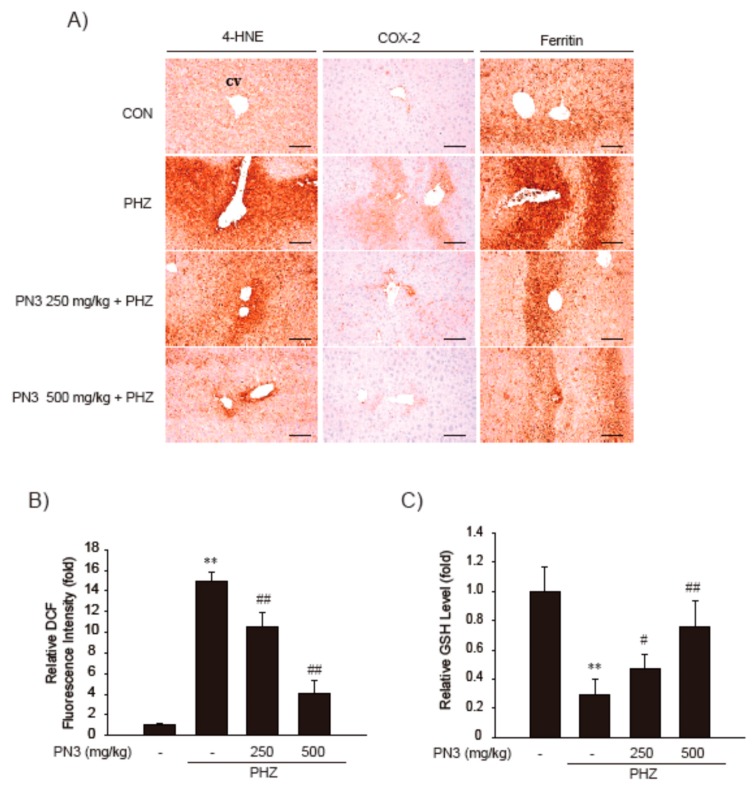
The effect of the ethyl acetate fraction PN3 on PHZ-induced liver injury. (**A**) The observation of 4-HNE, COX-2, and ferritin immunostained hepatocytes. (**B**) Determination of ROS generation in mice liver. After frozen livers were stained with 5 μM DCFH-DA, ROS levels were measured by a fluorescence microplate reader. (**C**) Measurement of GSH. GSH concentration was measured in mouse liver homogenates. Data represent the mean ± S.D.; ** *p* < 0.01, significant versus vehicle-treated control; ^#^ *p* < 0.05, ^##^ *p* < 0.01, significant versus PHZ alone.

**Figure 8:**
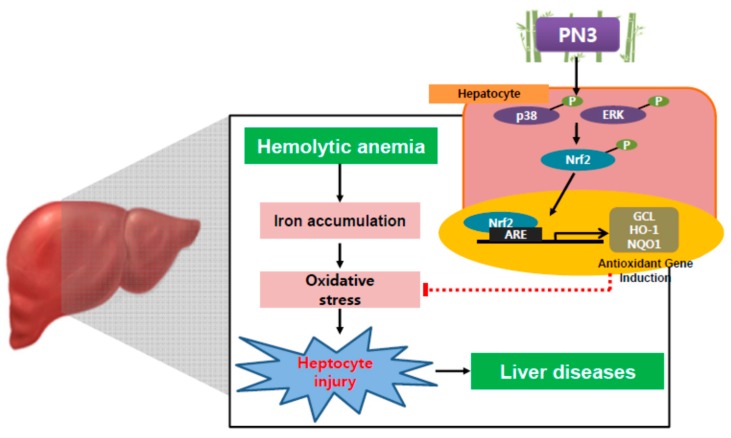
Schematic diagram illustrating the mechanism by which the PN3 protected the cells against iron overload-induced liver injury.

Immunohistochemistry of hepatic tissues showed that numbers of cells that were 4-HNE (a lipid peroxidation marker)-, COX-2 (inflammatory response marker)-, and ferritin (hepatic iron accumulation marker)-positive increased after PHZ administration, indicating that PHZ induces oxidative stress and inflammatory responses via iron accumulation in hepatic tissues. However, these PHZ-induced changes were significantly inhibited by PN3 administration (Figure 7A and Table 1). Next, we measured ROS levels in liver tissues in each group of mice using DCFH-DA dye. Similarly, PHZ-treated mice displayed a significant increase in the ROS levels as compared to those observed in vehicle-treated control mice. However, administration of PN3 obviously reduced ROS levels in liver tissue (Figure 7B). Similarly, PN3 administration restored the GSH content that was depleted by PHZ treatment (Figure 7C). These results propose that PN3 protects hepatic tissues from PHZ-mediated oxidative stress (Figure 8).

## 4. Discussion

We have previously characterized major active components of a domestic bamboo species, *P. nigra* var. heronis, to report the anti-oxidative and anti-melanogenic effects of its stem on melanocytes [23]. However, pharmacological properties and mechanism of action of bamboo stems remain poorly understood. In the current study, we showed that PN3 exerts a hepatoprotective effect against iron overload-induced acute liver injury due to its antioxidant properties.

It has been reported that Nrf2 serves as the master regulator of anti-oxidative response, which leads to an increased expression of a battery of oxidative stress response proteins and enzymes associated with detoxification and glutathione biosynthesis [32]. Although Nrf2 is ubiquitously expressed, it is especially considered to be a potential therapeutic target to prevent and/or treat liver injury [15,33]. Here, we compared Nrf2 activities using an *NQO1-*ARE firefly luciferase construct, which contained three tandem repeats of the ARE in the 5′-upstream region of NQO1, from the stem extracts of two species of bamboo, *P. bambusoides* and *P. nigra* var. heronis, under varying extraction conditions. We found that extract from *P. nigra* var. heronis is more potent than *P. bambusoides* in Nrf2 activation and PN3 has a much higher luciferase activity (Figure 1). Furthermore, PN3 activates Nrf2 and its downstream genes through stimulation of ARE-binding activity. The activation of Nrf2 by PN3 was observed to be regulated by upstream kinases, including ERK, p38 MAPK, or PKCδ (Figure 3B,C).

Oxidative stress plays a major role in acute and chronic liver injury. In hepatocytes, oxidative stress frequently triggers antioxidant response by activating Nrf2, which upregulates various cytoprotective genes. Various hepatocyte cell lines and primary hepatocytes have been used to demonstrate mechanisms of phytochemicals-induced Nrf2 activation. A few polyphenols and their derivatives (eckol, quercetin, phloretamide, and epicatechin) have been reported to activate Nrf2 signaling in hepatocytes even in the absence of exogenous oxidative stress stimuli [34,35]. Sharma et al. reported the anti-oxidant and hepatoprotective effect of polyphenols via apoptosis inhibition and Nrf2 activation in mice [36].

To determine the efficacy of PN3 and its major polyphenols against oxidative damage, we treated HepG2 cells with AA + iron. The level of iron in the liver is tightly regulated and an elevation of the iron level in liver results in excess ROS formation, which can then lead to the development of liver diseases, such as steatohepatitis, fibrosis, cirrhosis, and even hepatocellular carcinoma [2,6]. The release of AA, a component of membrane phospholipids, increased by oxidative stress, including high levels of iron [37]. We previously established AA + iron-induced cell injury model, which was useful for the identification of drug candidates against oxidative stress-mediated cell damage [12,38]. We further investigated the effects of PN3 and its major constituent polyphenolson iron + AA-induced oxidative stress and cell death in HepG2 cells. Here, we observed that PN3 and its major constituent polyphenols such as catechin, chlorogenic acid, caffeic acid, and *p*-coumaric acid succeeded in preventing ROS generation and the depletion of glutathione level, and thus, helped confirm their relation to cytotoxicity (Figure 4 and Figure 5). There might be other compounds than the primary polyphenols in PN3. Therefore, extensive works on characterization and validation of the extracts for food and cosmetic application are underway.

Treatment with PHZ led to hemolytic anemia and caused excess iron accumulation in liver, resulting in hepatocyte death and increased plasma AA [30]. Thus, PHZ-induced mouse model of acute liver injury was employed to confirm our in vitro finding that PN3 protects cells from AA + iron. As previously reported, ALT and AST levels increased due to PHZ administration, which were then attenuated by administration of PN3. Furthermore, H and E staining showed that hepatocyte degeneration and inflammatory cell were increased in the PHZ-treatment group; these changes were prohibited by PN3 treatment through inhibition of lipid peroxidation, inflammatory response, and iron accumulation in the liver (Figure 6 and Figure 7). Although H and E staining was generally used to analyze damage and/or inflammation based on the size and shape of the liver tissue, special stains to identify specific inflammatory cells was still required.

In this study, PN3 (containing a polyphenol mixture) was observed to function as an Nrf2 inducer, resulting in regulation of redox reactions in vivo. Recently, antioxidant activity from natural dietary compounds was proven to be associated with redox homeostasis [39]. Redox homeostasis may be defined as the internal dynamic equilibrium with respect to the continuous alterations of electrophilic and nucleophilic tones that helps maintain the optimum redox steady state [40]. The maintenance of redox homeostasis is crucial for preserving physiological functions since ROS/RNS are constantly generated in the normal metabolism of aerobic cells [41]. The fine regulation of the dynamic equilibrium between electrophilic and nucleophilic tones is at the base of redox homeostasis. Recently, chlorogenic acid was reported to be a promising therapeutic agent for the detoxification of acetaminophen-induced hepatotoxicity. Wei et al. reported that chlorogenic acid protects against APAP-induced hepatotoxicity by activating Nrf2 signaling pathway by blocking the binding of Nrf2 to its inhibitor protein, Keap1; furthermore, ERK1/2 has been reported to play a critical role in regulating chlorogenic acid-induced Nrf2 transcriptional activation [42]. There have been many report one the pleiotropic effect of polyphenol mixture and nutrients combination against various cancers including liver cancer [43]. To the best of our knowledge, this is the first report on the effect of polyphenol mixture on liver protection. Much work should be done to extend our understanding of the mechanism of polyphenol mixture of any physiological and therapeutic relevance. Many polyphenol-rich foods, including fruits, vegetables, and herbs, have been widely consumed for various health purposes, including as antioxidants to improve liver diseases, although their polyphenolic content and in vivo hepato-recovery effects have yet to be evaluated. In addition to fresh foods, fermented ones have been reported to have in vivo hepato-recovery effects [44]. In this regard, it is plausible that bamboo trees and their extracts could act as therapeutic agents for the prevention and/or treatment of liver diseases.

In conclusion, PN3 extract, containing a mixture of the above polyphenols, activates Nrf2 and protects against iron overload-mediated cell death in HepG2 cells. Major constituent polyphenols exert cytoprotective effects in HepG2 cells, along with anti-oxidative effects. We also demonstrated in this study that PN3 protects liver against iron overload-induced acute liver injury animal model. These findings suggested that PN3 may be a promising therapeutic agent for the prevention of acute liver injury. Continuing work on the effect of validated bamboo extract on other physiological activities are under way.

## Figures and Tables

**Table 1:** Histomorphometrical analysis of hepatic tissues, taken form vehicle or PHZ-treated mice.

	Groups	Vehicle	PHZ	PHZ + PN3 250 mg/kg	PHZ + PN3 500 mg/kg
Index (Unit)					
DE regions (%/mm^2^)		2.69 ± 2.21	59.93 ± 12.69 **	36.17 ± 11.09 ^##^	11.00 ± 3.50 ^##^
DE hepatocytes (cells/1000 hepatocytes)		32.50 ± 25.62	587.50 ± 107.89 **	297.33 ± 115.40 ^##^	99.33 ± 26.44 ^##^
Inflammatory cells (cells/mm^2^)		23.83 ± 14.66	196.00 ± 93.34 **	92.83 ± 24.73 ^##^	63.67 ± 21.92 ^##^
Immunopositive cells (cells/1000 hepatocytes)					
4-Hydroxynonenal		42.00 ± 21.16	608.33 ± 179.78 **	265.00 ± 73.54 ^##^	95.67 ± 19.29 ^##^
Cyclooxygenase-2		3.67 ± 3.27	324.17 ± 143.76 **	88.83 ± 19.77 ^##^	42.17 ± 23.16 ^##^
Ferritin		75.67 ± 29.38	566.17 ± 123.01 **	288.50 ± 78.42 ^##^	141.67 ± 50.25 ^##^

Values are expressed as mean ± SD of six histological fields (** *p* < 0.01 as compared to those of vehicle treatment or ^##^ *p* < 0.01 as compared to those of PHZ treatment); DE = Degenerated.

## Supplementary Materials

The following are available online at http://www.mdpi.com/2072-6643/11/1/114/s1, Figure S1: Cell viability due to *P. bambusoides* (PB) and *P. nigra* var. henonis (PN) extracts in HepG2 cells. (**A** and **B**): HepG2 cells were treated with extracts of PB (PB1-5: 100 µg/mL) and PN (PN1-5: 100 µg/mL) for 24 h. Data represent the mean ± S.D. of 3 separate experiments, where the statistical significance of differences between each treatment group and the vehicle-treated control are given.
