# Supplementary material for: Bamboo Stems (Phyllostachys nigra variety henosis) Containing Polyphenol Mixtures Activate Nrf2 and Attenuate Phenylhydrazine-Induced Oxidative Stress and Liver Injury
Source: Nutrients. 2019 Jan 8;11(1):114. doi: 10.3390/nu11010114 (PMC6357197; doi:10.3390/nu11010114)
Supplement: Supplementary file 1 [file nutrients-11-00114-s001.pdf]

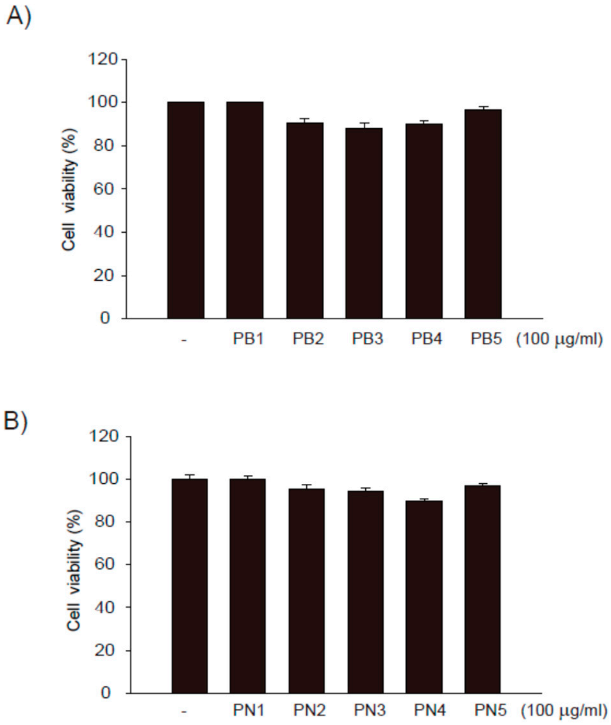

Supplementary Figure 1. Cell viability due to *Phyllostachys bambusoides* (PB) and *Phyllostachys nigra* var. henonis (PN) extracts in HepG2 cells.

A and B, HepG2 cells were treated with extracts of PB (PB1-5: 100 µg/mL) and PN (PN1-5: 100 µg/mL) for 24 h. Data represent the mean ± S.D. of 3 separate experiments, where the statistical significance of differences between each treatment group and the vehicle-treated control are given.
